# An update on overweight and obesity in rural Northeast China: from lifestyle risk factors to cardiometabolic comorbidities

**DOI:** 10.1186/1471-2458-14-1046

**Published:** 2014-10-08

**Authors:** Xiaofan Guo, Zhao Li, Liang Guo, Liqiang Zheng, Shasha Yu, Hongmei Yang, Lu Zou, Ying Zhou, Yaowen Zhang, Luoning Zhu, Yonghong Zhang, Yingxian Sun

**Affiliations:** Department of Cardiology, the First Hospital of China Medical University, 155 Nanjing North Street, Heping District, Shenyang, Liaoning 110001 People’s Republic of China; Department of Clinical Epidemiology, Library, Shengjing Hospital of China Medical University, Shenyang, Liaoning People’s Republic of China; Department of Cardiology, Dongfang Hospital affiliated to Tongji University, Shanghai, People’s Republic of China; Department of Epidemiology, School of Public Health, Medical College of Soochow University, Suzhou, Jiangsu People’s Republic of China

**Keywords:** General obesity, Abdominal obesity, Prevalence, Risk factor, Comorbidities

## Abstract

**Background:**

Not enough is known about the prevalence of overweight and obesity in rural China in the current decade. We aim to update our knowledge of the prevalence of obesity and its associated risk factors and comorbidities in a large population sample in rural Northeast China.

**Methods:**

A population-based survey of 11,579 participants aged 35 years and older was conducted in rural areas of Liaoning Province during 2012–2013. Anthropometric measurements, information on health-related variables and blood biochemical indexes were collected by well-trained personnel.

**Results:**

The prevalence of general obesity and overweight was found to be 7.8% and 37.2%, respectively. The overall prevalence of abdominal obesity was 15.1%. Female gender, ethnic minority, middle-school education and a family income of 5,000–20,000 CNY per year were found to be risk factors for general obesity, while older age, female gender, ethnic minority and longer sleep duration (>8 h/d) increased the risk of abdominal obesity, after adjusting for confounders. Overweight and obese participants had significantly higher risks to develop prehypertension, hypertension, high LDL-C and low HDL-C compared with normal weight participants, while abdominal obesity was associated with increased risks of diabetes and high TG after adjusted for multiple factors. Compared with participants with a normal BMI and no abdominal obesity, the participants classified as abdominally obese and normal BMI; as abdominally obese and overweight; and abdominally obese and generally obese each had a progressive increase in the odds of hypertension (OR: 1.961, 95% CI: 1.154 to 3.331, OR: 2.744, 95% CI: 2.126 to 3.541, and OR: 8.990, 95% CI: 5.858 to 13.795, respectively) and high TG (OR: 3.165, 95% CI: 2.183 to 4.588, OR: 3.980, 95% CI: 3.332 to 4.755, and OR: 4.340, 95% CI: 3.574 to 5.271, respectively).

**Conclusions:**

The prevalence of obesity in rural Northeast China exhibited a remarkably increasing upwards trend. General and abdominal obesity were associated with different subtypes of cardiometabolic comorbidities, the combined effects of which on the comorbidities dramatically increased.

## Background

The prevalence of overweight and obesity has been increasing rapidly all over the world [1, 2], posing a major public health burden worldwide. In developing countries, rates of obesity have tripled in the past 20 years [3]. Aside from being a potentially modifiable cardiovascular disease (CVD) risk factor on its own, this non-communicable disease has propelled an upsurge in other cardiometabolic comorbidities, including hypertension, dyslipidaemia, Type 2 diabetes and metabolic syndrome [3, 4].

The frequently-used anthropometric measurements of adiposity are body mass index (BMI) and waist circumference (WC). Abdominal fat deposition measured by WC has been suggested as a better indicator of obesity in relation to CVDs than BMI [5, 6]. Because BMI reflects a combination of both fat mass and lean mass and WC reflects a measure of central fat distribution, the combination of these two indexes might be a more powerful predictor for health risks. Incorporating evaluation of WC in addition to BMI in clinical practice was advocated by many previous studies [7, 8]. However, there have been limited studies clarifying the associations between these two parameters and cardiometabolic comorbidities, such as hypertension, diabetes and dyslipidaemia, in the rural Chinese population.

As in other developing countries, overweight and obesity have become an important public health problem in China. In China in 2000–2001 the prevalence of overweight reached 26.9% in men and 31.1% in women [9]. Our previous study conducted during 2004–2005 found that in rural Northeast China the overall prevalence of overweight was 18.6% [10]. Rural China is experiencing both a rapid increase in economic progress and an epidemiologic transition. The prevalence of overweight and obesity in rural areas after the year 2010 is unknown. The Northeast area has its own climate, geography, history and lifestyles that differ from those of other regions of China. We hypothesized that huge changes may have taken place in the prevalence, risk factors and cardiometabolic comorbidities of obesity in rural Northeast China during this 10-year period. Therefore, we undertook the present study to update our knowledge of the prevalence of general and abdominal obesity, as well as their risk factors, in rural China; and to evaluate the independent and the combined effects of general and abdominal obesity on different comorbidities in a large sample size of the rural population during 2012–2013.

## Methods

### Study population

Liaoning Province is located in Northeast China with a population of more than 40 million. From January 2012 to August 2013, a large scale epidemiological study was conducted to evaluate the prevalence, incidence and natural history of cardiovascular risk factors in rural areas of Liaoning Province, which has around 40% of the total population residing in more than 20 counties. We used a random representative sample of the general rural population aged ≥35 years. The study adopted a multi-stage, stratified and random cluster-sampling design. In the first stage, 3 counties (Dawa, Zhangwu, and Liaoyang County) were selected from the Eastern, Southern, and Northern regions of Liaoning province. In the second stage, 1 town was randomly selected from each county (a total of 3 towns). In the third stage, 8–10 rural villages from each town were randomly selected, yielding a total of 26 rural villages included in the study. All eligible permanent residents aged ≥35 years from each village were invited to enroll in the study (a total of 14,016 participants). Of those, 11,956 participants with a mean ± standard value (SD) of 53.9 ± 10.6 years agreed and completed the study, yielding an overall response rate of 85.3%. Participants with pregnancy, malignant tumor and mental disorder were excluded from the study. This study was approved by the Ethics Committee of China Medical University (Shenyang, China). All procedures were performed in accordance with the institution’s ethical standards. Written consent was obtained from all participants after they had been informed of the objectives, benefits, medical particulars and confidentiality agreements for personal information. If the participants were illiterate, we obtained written informed consent from their proxies. In this report, we used data of baseline and only participants with a complete set of data regarding the variables analyzed in the study was included, making a final sample size of 11,579 (5,361 men and 6,218 women).

### Data collection and measurements

Data collection was performed in the local healthcare centers in participants’ residential area. Data were collected by cardiologists and trained nurses using a standard questionnaire delivered by face-to-face interview. Before the survey was performed, all eligible investigators were invited to attend organized training. The training contents included the purpose of this study, how to administer the questionnaire, the standard method of measurement, the importance of standardization, and the study procedures. A strict evaluation was done after this training, and only those who scored perfectly on the test could become investigators. During data collection, our inspectors received further instructions and support.

Data on demographic characteristics, lifestyle risk factors, dietary habits, family income, family history of cardiovascular disease, medical history of hypertension, evaluation of psychological status, and quality of life were obtained by interview using a standardized questionnaire. A central steering committee with a subcommittee was established for the purposes of quality control. Educational level was divided into primary school or below, middle school, and high school or above. Family income was classified as ≤5,000, 5,000–20,000 and >20,000 CNY/year. Smoking and drinking status were classified by the participants’ self-report as current versus nonsmokers. Self-reported sleep duration (including nocturnal and nap duration) was obtained from the questionnaire. The responses were categorized into four groups: ≤7, 7–8, 8–9, and >9 h/d.

All anthropometric measurements were made by two trained cardiologists. According to the American Heart Association protocol, blood pressure was measured 3 times at 2-min intervals after at least 5 min of rest using a standardized automatic electronic sphygmomanometer (HEM-907; Omron, Tokyo, Japan), which had already been validated according to the British Hypertension Society protocol [11]. The participants were advised to avoid caffeinated beverages and exercise for at least 30 min before the measurement. During the measurement, the participants were seated with the arm supported at the level of the heart. The mean of 3 blood pressure (BP) measurements was calculated and used in all analyses.

Weight was measured with the participants wearing lightweight clothing on a calibrated scale and height was measured without shoes using a portable stadiometer. Weight and height were measured to the nearest 0.1 kg and 0.1 cm respectively. WC was measured at the umbilicus using a non-elastic tape (to the nearest 0.1 cm), with the participants standing at the end of normal expiration. BMI was calculated as weight in kilograms divided by the square of the height in meters.

Fasting blood samples were collected in the morning after at least 12 h of fasting for all participants. Blood samples were obtained from an antecubital vein into Vacutainer tubes containing EDTA. Serum was subsequently isolated from the whole blood, and all serum samples were frozen at -20°C for testing at a central certified laboratory. Fasting plasma glucose (FPG), total cholesterol (TC), low-density lipoprotein cholesterol (LDL-C), high-density lipoprotein cholesterol (HDL-C), triglyceride (TG) and other routine blood biochemical indexes were analyzed enzymatically on an Olympus AU640 auto analyzer (Olympus, Kobe, Japan). All analytes were measured in an auto analyzer (Bayer RA-XT, Tarrytown, NY, USA) using kits by the same company.

### Definitions

According to the JNC-7 report [12], prehypertension was defined as not being on antihypertensive medication and having a systolic BP (SBP) of 120–139 mmHg or diastolic BP (DBP) of 80–89 mmHg. Hypertension was defined as SBP ≥ 140 mmHg and/or DBP ≥ 90 mmHg and/or use of antihypertensive medications. BMIs were categorized into 3 groups as normal (BMI < 25 kg/m^2^), overweight (25 ≤ BMI <30 kg/m^2^) and obesity (BMI ≥ 30 kg/m^2^), according to the World Health Organization (WHO) criteria [13]. Abdominal obesity was defined as WC ≥ 88 cm for females and WC ≥ 102 cm for males [14]. Dyslipidemia was defined according to the National Cholesterol Education Program-Third Adult Treatment Panel (ATP III) criteria [15]. High TC was defined as TC ≥ 6.21 mmol/L(240 mg/dL). Low HDL-C was defined as HDL-C < 1.03 mmol/L (40 mg/dL). High LDL-C was defined as LDL-C ≥ 4.16 mmol/L (160 mg/dL). High TG was defined as ≥2.26 mmol/L (200 mg/dL). Diabetes mellitus was diagnosed according to the WHO criteria: FPG ≥ 7 mmol/L (126 mg/dL) and/or being on treatment for diabetes [16].

Physical activity included occupational and leisure-time physical activity. A detailed description of the methods has been presented elsewhere [17]. Occupational and leisure-time physical activity were merged and regrouped into 3 categories: (1) low—subjects who reported light levels of both occupational and leisure-time physical activity; (2) moderate—subjects who reported moderate or high levels of either occupational or leisure-time physical activity; and (3) high—subjects who reported a moderate or high level of both occupational and leisure-time physical activity.

The dietary pattern was assessed using the subjects’ recall of foods eaten in the previous year. The questionnaire included questions on the average consumption of several food items per week. The reported consumption was quantified approximately in terms of grams per week (vegetable consumption: rarely = 3, <1,000 g = 2, 1,000–2,000 g = 1, ≥2,000 g = 0; meat consumption including red meat, fish, and poultry: rarely = 0, <250 g = 1, 250-500 g = 2, ≥500 g = 3). A special diet score (vegetable consumption score plus meat consumption score) was calculated for each participant (range 0–6). Higher values of the diet score indicated higher meat consumption and lower vegetable consumption and greater adherence to a Westernized diet, while lower values indicated adherence to the Chinese-diet. Similar methods for calculating diet score could be found in the ATTICA study [18].

### Statistical analysis

Descriptive statistics were calculated for all the variables, including continuous variables (reported as mean values and SDs) and categorical variables (reported as numbers and percentages). Differences among weight categories were evaluated using Student’s t-test, ANOVA, non-parametric test or the χ2-test as appropriate. Multivariate logistic regression analyses were used to identify independent factors and associated comorbidities of general obesity and abdominal obesity with odds ratios (ORs) and corresponding 95% confidence intervals (CIs) calculated. All the statistical analyses were performed using SPSS version 17.0 software, and *P* values less than 0.05 were considered to be statistically significant.

## Results

### Baseline characteristics of study population

Table 1 presents the baseline characteristics of included participants according to BMI and WC categories. Older participants were more likely to have normal weight and to be abdominally obese. A higher proportion of either general obesity or abdominal obesity was observed among females. Ethnic difference was only observed in BMI categories. Participants with general or abdominal obesity tended to have a more unfavorable metabolic profile (eg. higher values of SBP, DBP, FPG, TC, TG and LDL-C, all *P* < 0.05). In addition, current smokers or drinkers were more common in participants with normal BMI and WC.

**Table 1:** **Baseline characteristics of study population according to weight categories**

Variables	BMI (kg/m ^2^)				WC (cm)		
	Normal weight	Overweight	Obesity	***P***-value	Non-obesity	Abdominal obesity	***P***-value
**Age (year)**	54.3 ± 10.9	53.5 ± 10.2	52.3 ± 9.9	<0.001	53.6 ± 10.6	54.9 ± 10.1	<0.001
**Male gender**	3010 (47.3)	1997 (46.3)	354 (39.4)	<0.001	5151 (52.4)	210 (12.0)	<0.001
**Ethnicity**							
Han	6069 (95.3)	4072 (94.5)	833 (92.7)		9330 (94.9)	1644 (94.0)	
Others^a^	300 (4.7)	239 (5.5)	66 (7.3)	0.002	500 (5.1)	105 (6.0)	0.112
**Education**							
Primary school or below	3183 (50.0)	2162 (50.2)	422 (46.9)		4719 (48.0)	1048 (59.9)	
Middle school	2612 (41.0)	1730 (40.1)	377 (41.9)		4147 (42.2)	572 (32.7)	
High school or above	574 (9.0)	419 (9.7)	100 (11.1)	0.151	964 (9.8)	129 (7.4)	<0.001
**Physical activity**							
Low	1814 (28.5)	1353 (31.4)	276 (30.7)		2789 (28.4)	654 (37.4)	
Moderate	4213 (66.1)	2700 (62.6)	570 (63.4)		6507 (66.2)	976 (55.8)	
High	342 (5.4)	258 (6.0)	53 (5.9)	0.006	534 (5.4)	119 (6.8)	<0.001
**Family income (CNY/year)**							
≤5000	871 (13.7)	484 (11.2)	87 (9.7)		1213 (12.3)	229 (13.1)	
5000-20000	3430 (53.9)	2371 (55.0)	509 (56.6)		5319 (54.1)	991 (56.7)	
>20000	2068 (32.5)	1456 (33.8)	303 (33.7)	<0.001	3298 (33.6)	529 (30.2)	0.025
**Current smoking status**	2525 (39.6)	1342 (31.1)	222 (24.7)	<0.001	3765 (38.3)	324 (18.5)	<0.001
**Current drinking status**	1490 (23.4)	958 (22.2)	170 (18.9)	0.008	2480 (25.2)	138 (7.9)	<0.001
**Diet score**	2.3 ± 1.1	2.4 ± 1.1	2.3 ± 1.1	<0.001	2.4 ± 1.1	2.2 ± 1.2	<0.001
**Sleep duration (h/d)**	7.2 ± 1.7	7.3 ± 1.7	7.4 ± 1.6	<0.001	7.3 ± 1.7	7.2 ± 1.7	0.437
**SBP (mmHg)**	137.7 ± 22.6	145.9 ± 23.4	150.8 ± 23.5	<0.001	140.4 ± 22.9	149.3 ± 25.1	<0.001
**DBP (mmHg)**	79.8 ± 11.3	84.4 ± 11.6	87.2 ± 12.0	<0.001	81.5 ± 11.6	85.0 ± 12.0	<0.001
**BMI (kg/m** ^**2**^ **)**	22.2 ± 1.9	27.1 ± 1.4	32.4 ± 2.9	<0.001	24.0 ± 3.1	29.2 ± 3.4	<0.001
**WC (cm)**	76.7 ± 7.1	87.6 ± 6.6	98.3 ± 8.3	<0.001	80.1 ± 8.4	95.3 ± 7.0	<0.001
**TC (mmol/L)**	5.1 ± 1.1	5.3 ± 1.1	5.4 ± 1.2	<0.001	5.2 ± 1.1	5.5 ± 1.2	<0.001
**TG (mmol/L)**	1.4 ± 1.3	1.9 ± 1.6	2.2 ± 2.0	<0.001	1.5 ± 1.4	2.1 ± 1.6	<0.001
**LDL-C (mmol/L)**	2.8 ± 0.8	3.1 ± 0.8	3.2 ± 0.9	<0.001	2.9 ± 0.8	3.2 ± 0.9	<0.001
**HDL-C (mmol/L)**	1.5 ± 0.4	1.3 ± 0.3	1.3 ± 0.3	<0.001	1.4 ± 0.4	1.3 ± 0.3	<0.001
**FPG (mmol/L)**	5.7 ± 1.5	6.1 ± 1.8	6.3 ± 1.8	<0.001	5.8 ± 1.6	6.3 ± 2.0	<0.001

Data are expressed as the mean ± SD or as n (%).

*Abbreviations:* *BMI* body mass index, *WC* waist circumference, *CNY* China Yuan (1CNY = 0.161 USD), *SBP* systolic blood pressure, *DBP* diastolic blood pressure, *TC* total cholesterol, *TG* triglyceride, *LDL-C* low-density lipoprotein cholesterol, *HDL-C* high-density lipoprotein cholesterol, *FPG* fasting plasma glucose.

^a^Including some ethnic minorities in China, such as Mongol and Manchu.

### Prevalence of overweight, obesity and abdominal obesity

Table 2 presents the prevalence of overweight, general obesity and abdominal obesity among the different groups. Mean BMI and WC were 24.8 ± 3.7 kg/m^2^ and 82.4 ± 9.8 cm, respectively. The overall prevalence of general obesity and overweight was 7.8% (6.6% for males and 8.8% for females) and 37.2% (37.3% for males and 37.2% for females), respectively. The overall prevalence of abdominal obesity was 15.1% (3.9% for males and 24.8% for females). The distribution of obesity status differed among four age categories.

**Table 2:** **Prevalence of overweight, obesity and adominal obesity in rural Chinese population by age and sex**

Age (year)	Sample size	BMI (kg/m ^2^)	Overweight	Obesity	WC (cm)	Abdominal obesity
**Male**						
35-44	1219	25.4 ± 4.0	468 (38.4)	124 (10.2)	84.8 ± 10.2	71 (5.8)
45-54	1621	24.9 ± 3.3	648 (40.0)	108 (6.7)	84.0 ± 9.4	62 (3.8)
55-64	1636	24.4 ± 3.4	584 (35.7)	85 (5.2)	83.2 ± 9.5	52 (3.2)
≥65	885	24.0 ± 3.4	297 (33.6)	37 (4.2)	83.2 ± 10.0	25 (2.8)
All	5361	24.7 ± 3.5	1997 (37.3)	354 (6.6)	83.8 ± 9.8	210 (3.9)
**Female**						
35-44	1533	24.8 ± 3.8	538 (35.1)	125 (8.2)	79.2 ± 9.1	256 (16.7)
45-54	1978	25.1 ± 3.6	783 (39.6)	190 (9.6)	81.4 ± 9.2	491 (24.8)
55-64	1848	25.0 ± 3.8	690 (37.3)	171 (9.3)	82.5 ± 10.0	537 (29.1)
≥65	859	24.2 ± 3.9	303 (35.3)	59 (6.9)	82.1 ± 10.8	255 (29.7)
All	6218	24.9 ± 3.8	2314 (37.2)	545 (8.8)	81.3 ± 9.7	1539 (24.8)

Data are expressed as the mean ± SD or as n (%).

*Abbreviations:* *BMI* body mass index, *WC* waist circumference.

### Factors associated with obesity status

Table 3 presents the multivariable logistic regression analysis of risk factors related to general and abdominal obesity. Increased age was associated with an increased risk of abdominal obesity but a decreased risk of general obesity (both *P* for trend <0.001). Compared with males, females were at greater risk of developing both general obesity (OR: 3.777, 95% CI: 2.958 to 4.822) and abdominal obesity (OR: 7.297, 95% CI: 6.089 to 8.744). Ethnic minority was associated with a higher risk of both types of obesity compared to the ethnicity of Han (general obesity: OR: 1.469, 95% CI: 1.020 to 2.114; abdominal obesity: OR: 1.299, 95%CI: 1.031 to 1.638). Current smokers were less likely to be generally or abdominally obese. Moderate physical activity was found to be a protective factor for abdominal obesity (OR: 0.858, 95% CI: 0.762 to 0.966), while longer sleep durations (>8 h/d) increased its risk. The OR of general obesity was increased (*P* < 0.05) for those with a family income of 5,000–20,000 CNY per year in comparison to those with a lower family income (≤5,000 CNY per year). Education at middle school had opposite effects on the two obesity types.

**Table 3:** **Multiple regression analyses of general and abdominal obesity and associated factors**

Variables	General obesity		***P***-value for trend	Abdominal obesity		***P***-value for trend
	OR (95% CI)	***P***-value		OR (95% CI)	***P***-value	
**Age (year)**			<0.001			<0.001
35-44	1.000 (reference)			1.000 (reference)		
45-54	0.76 (0.597-0.967)	0.026^*^		1.379 (1.182-1.610)	<0.001^*^	
55-64	0.503 (0.384-0.658)	<0.001^*^		1.536 (1.301-1.814)	<0.001^*^	
≥65	0.338 (0.236-0.483)	<0.001^*^		1.457 (1.187-1.788)	<0.001^*^	
**Gender**						
Male	1.000 (reference)			1.000 (reference)		
Female	3.777 (2.958-4.822)	<0.001^*^		7.297 (6.089-8.744)	<0.001^*^	
**Ethnicity**						
Han	1.000 (reference)			1.000 (reference)		
Others^a^	1.469 (1.020-2.114)	0.039^*^		1.299 (1.031-1.638)	0.027^*^	
**Education**			0.023			0.014
Primary school or below	1.000 (reference)			1.000 (reference)		
Middle school	1.249 (1.010-1.544)	0.04^*^		0.856 (0.754-0.972)	0.017^*^	
High school or above	1.382 (0.995-1.918)	0.053		0.837 (0.675-1.038)	0.104	
**Physical activity**			0.113			0.516
Low	1.000 (reference)			1.000 (reference)		
Moderate	1.211 (0.980-1.496)	0.077		0.858 (0.762-0.966)	0.011^*^	
High	1.130 (0.759-1.682)	0.548		1.150 (0.914-1.448)	0.234	
**Family income (CNY/year)**			0.347			0.251
≤5000	1.000 (reference)			1.000 (reference)		
5000-20000	1.438 (1.042-1.982)	0.027^*^		0.967 (0.815-1.148)	0.702	
>20000	1.354 (0.960-1.910)	0.084		0.883 (0.731-1.067)	0.197	
**Current smoking status**	0.678 (0.540-0.852)	0.001^*^		0.724 (0.627-0.836)	<0.001^*^	
**Current drinking status**	0.897 (0.680-1.182)	0.439		1.074 (0.861-1.339)	0.529	
**Sleep duration (h/d)**			0.737			0.002
≤7	1.000 (reference)			1.000 (reference)		
7-8	1.037 (0.835-1.289)	0.741		1.062 (0.934-1.208)	0.361	
8-9	0.944 (0.717-1.243)	0.681		1.246 (1.058-1.467)	0.008^*^	
>9	1.017 (0.727-1.422)	0.923		1.335 (1.089-1.635)	0.005^*^	
**Diet score (per 1 unit)**	0.973 (0.896-1.057)	0.52		0.997 (0.950-1.047)	0.912	

*P < 0.05 for the independent association between general obesity or abdominal obesity and each factor after adjusting for the remaining factors.

*Abbreviations:* *OR* odds ratio, *95% CI* 95% confidence interval, *CNY* China Yuan (1CNY = 0.161 USD).

^a^Including some ethnic minorities in China, such as Mongol and Manchu.

### Weight status and cardiometabolic comorbidities

**Figure 1:**
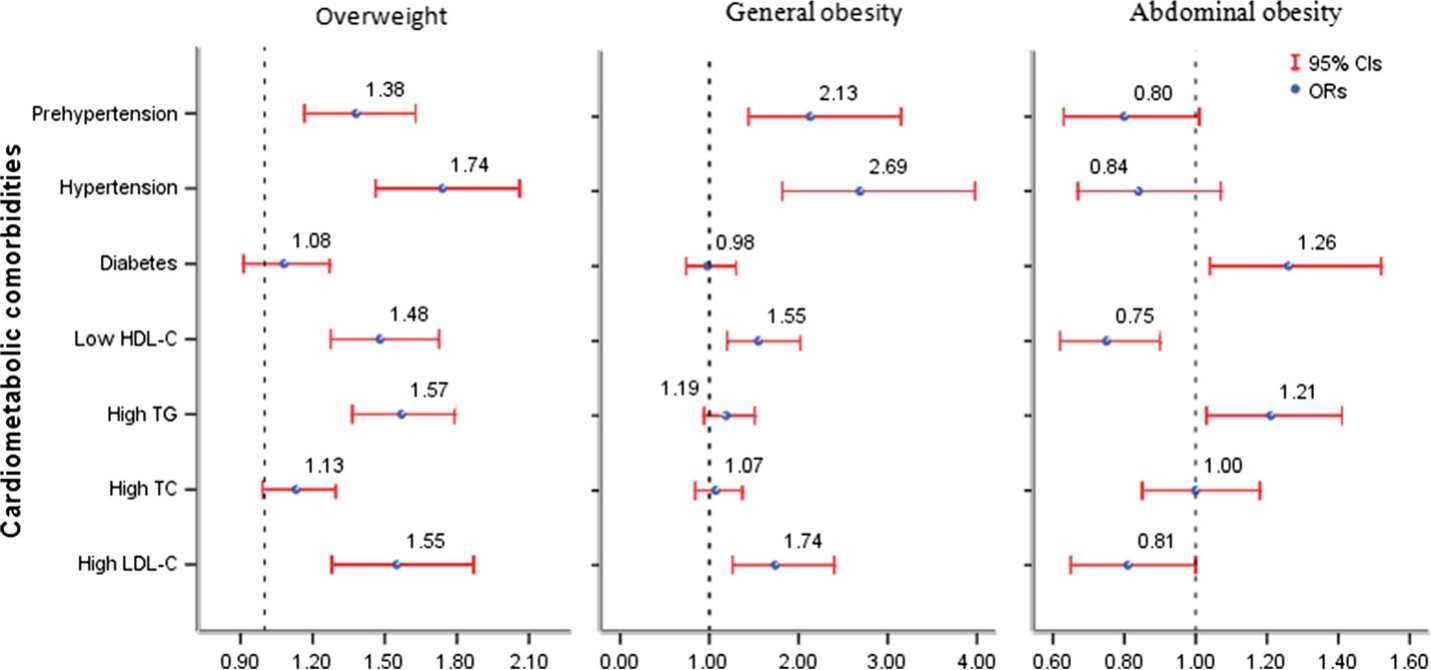
**Associations between weight status and different cardiometabolic comorbidities.** Results were adjusted for age, gender, ethnicity, education, physical activity, family income, smoking status, drinking status, sleep duration, diet, blood pressure, fasting plasma glucose and lipid status.

Figure 1 presents associations between weight status and cardiometabolic comorbidities. Overweight and obese participants had significantly higher risks for prehypertension, hypertension, high LDL-C and low HDL-C compared with normal weight participants, while abdominal obesity was associated with increased risks of diabetes and high TG after adjustment for the potential contributions. Table 4 presents combined effects of general and abdominal obesity on the cardiometabolic comorbidities. Participants with both general and abdominal obesity had much higher risks of all seven kinds of comorbidities. Compared with participants with a normal BMI and no abdominal obesity, the participants classified as abdominally obese and normal BMI, as abdominally obese and overweight, and abdominally obese and generally obese, each had a progressive increase in the odds of hypertension (OR: 1.961, 95% CI: 1.154 to 3.331, OR: 2.744, 95% CI: 2.126 to 3.541, and OR: 8.990, 95% CI: 5.858 to 13.795, respectively) and high TG (OR: 3.165, 95% CI: 2.183 to 4.588, OR: 3.980, 95% CI: 3.332 to 4.755, and OR: 4.340, 95% CI: 3.574 to 5.271, respectively). With the exception of high TC, the odds for all the comorbidities were increased (*P* < 0.05) for those with solely general obesity compared with those with a normal BMI and no abdominal obesity.

**Table 4:** **Combined effects of general and abdominal obesity on different cardiometabolic comorbidities**

	Abdominal obesity + normal BMI		BMIAbdominal obesity + overweight		Abdominal obesity + general obesity		No abdominal obesity + general obesity	
	OR (95% CI)*	P-value	OR (95% CI)*	P-value	P-valueOR (95% CI)*	P-value	OR (95% CI)*	P-value
Prehypertension	1.169 (0.672-2.033)	0.582	1.330 (1.026-1.724)	0.031	3.714 (2.406-5.734)	<0.001	2.031 (1.146-3.597)	0.015
Hypertension	1.961 (1.154-3.331)	0.013	2.744 (2.126-3.541)	<0.001	8.990 (5.858-13.795)	<0.001	4.956 (2.836-8.661)	<0.001
Diabetes	1.175(0.726-1.903)	0.511	1.879 (1.515-2.330)	<0.001	1.949 (1.534-2.476)	<0.001	1.655 (1.113-2.459)	0.013
High TC	1.666 (1.160-2.393)	0.006	1.353 (1.128-1.624)	0.001	1.502 (1.221-1.847)	<0.001	1.319 (0.914-1.904)	0.14
Low HDL-C	2.388 (1.508-3.782)	<0.001	2.021 (1.609-2.538)	<0.001	3.010 (2.398-3.779)	<0.001	4.772 (3.543-6.429)	<0.001
High LDL-C	1.391 (0.830-2.332)	0.21	1.535 (1.196-1.971)	0.001	2.343 (1.806-3.041)	<0.001	1.853 (1.129-3.040)	0.015
High TG	3.165 (2.183-4.588)	<0.001	3.980 (3.332-4.755)	<0.001	4.340 (3.574-5.271)	<0.001	4.015 (2.988-5.395)	<0.001

*Adjusted for age, gender, ethnicity, education, physical activity, family income, smoking status, drinking status, sleep duration, diet, blood pressure, fasting plasma glucose and lipid statuswith participants with a normal BMI and no abdominal obesity as reference. *Abbreviations:* *BMI* body mass index, *OR* odds ratio, *95% CI* 95% confidence interval, *TC* total cholesterol, *TG* triglyceride, *LDL-C* low-density lipoprotein cholesterol, *HDL-C* high-density lipoprotein cholesterol.

## Discussion

The present study aimed at characterizing the current prevalence of overweight, obesity and abdominal obesity in rural areas of Northeast China. We found an increased prevalence of general obesity in both genders, and an especially high prevalence of abdominal obesity among females. General and abdominal obesity had different risk profiles and were associated with different comorbidities. The combined effects of both obesity types on cardiometabolic comorbidities was much larger than either one alone.

As a consequence of rapid economic development and industrialization, China has experienced rapidly escalating rates of obesity. It is estimated that the combined prevalence of overweight and obesity (BMI ≥ 25 kg/m^2^) increased by 49.5% between the years 1992 and 2002 [19]. During the period 1993–2009, the combined prevalence increased by 97%, from 13.4% in 1993 to 26.4% in 2009 [20]. In the present study, we found that the prevalence of general obesity and overweight rose around fourfold (8.8% vs. 2.2% for females and 6.6% vs.1.2% for males) and twofold (37.2% vs. 22.1% for females and 37.3% vs.15.1% for males), respectively, compared with 10 years ago [10]. Nearly half the population were generally obese or overweight, and the prevalence of abdominal obesity was approaching the rate in urban areas. This explosive growth might be explained by the fact that in recent years the rural population in Northeast China has experienced huge changes in lifestyle and an increase in life expectancy. The prevalence of general obesity and overweight we observed was higher than those in rural populations of other provinces in China [21] and other Asian countries, such as Japan and Korea [22, 23], using the same criteria. Also, we found that the prevalence was even higher than that in urban areas of the same province [24], challenging the viewpoint that overweight and obesity are more common in urban than in rural residents. The dramatic increase in the prevalence of obesity in rural areas was also observed in other countries [25, 26]. Taken together, the present study reinforces that the prevalence of overweight and obesity in the rural population in Northeast China has reached an alarming level, and in developing rural areas should become a public health concern.

Consistent with previous studies [24, 27, 28], we found that the risk of abdominal obesity increased with aging. However, interestingly, although evidence existed of a positive relation between aging and general obesity [29, 30], an inverse association was observed in the present population. This might be explained in part by the fact that aging caused a loss of subcutaneous fat, decrease of lean mass and accumulation of visceral fat [31–33], leading to a large proportion of the population developing abdominal obesity rather than general obesity. In developed countries, the prevalence of obesity is similarly high in both males and females. However, in countries with a relatively low gross national product, the prevalence of obesity is 1.5 to 2 times higher among females than among males [34]. In the present study, we found an especially higher prevalence of abdominal obesity among females, which was similar to the findings of other studies [35, 36]. For instance, Janghorbani et al. have reported that the prevalence of abdominal obesity in Iranian adults was found to be higher in women (53.5%) than in men (12.5%) [36]. Compared to males, we found that the risks for general obesity and abdominal obesity were about four times and seven times higher respectively in females. One possible explanation for the difference might be that in rural areas men engaged in more strenuous activity, and many women are mainly housewives, with less activity to engage in. However, the root causes for this gender-specific difference warrant further study. Unlike the results from urban areas of Liaoning province [24], we found that Han Chinese had a lower risk of obesity than other Chinese ethnic minorities. This might be attributed to genetic and lifestyle-related differences.

In line with the results of previous studies [36–38], an inverse association was observed between current smoking status and both general and abdominal obesity in the present study. This was further confirmed by the inverse dose–response relationship between the number of cigarettes smoked and overweight found by Xu et al. [39]. Smoking cessation might increase the risk of gaining overall body weight and developing central obesity [39], which should be taken into account when devising strategies for the prevention of obesity. In addition, we found that the effects of education level, physical activity, family income and sleep duration on general obesity and abdominal obesity were different. Further studies are expected to confirm these results.

The separate effects of general obesity or abdominal obesity on adverse cardiometabolic profile varied in a substantial number of epidemiologic studies, depending on the different populations, the confounding factors considered and the scales for measurement of variables. Our study found that general obesity was associated with a 2.1-fold and 2.7-fold risk for prehypertension and hypertension respectively, while abdominal obesity was associated with a 1.3-fold risk for diabetes. This could be explained by the observation that WC better reflected visceral fat and was closely associated with insulin resistance and diabetes, whereas BMI better indicated body volume and mass, which was associated with blood viscosity and blood volume, and was closely related to blood pressure [40]. To be noticed, in the present study some other endocrinological diseases such as type 1 diabetes, Cushing’s disease or hyperthyroidism that may cause body weight alteration were not excluded, which might compromise the results to some extent. We assumed that measuring WC could provide additional meaningful information beyond that provided by BMI for accurately predicting obesity-associated cardiometabolic comorbidities, which was supported by the results of our combined analyses of the two types of obesity. For instance, we found that the combination of general and abdominal obesity was associated with a much greater risk of hypertension compared with general obesity alone (ORs of 8.99 vs. 2.69). A similar result was also observed in a recent study based on the US National Health and Nutrition Examination Survey (NHANES) 2007–2010 [41]. Unlike the results from separate analysis, the risks of all the comorbidities, including prehypertension, hypertension, diabetes and four subtypes of dyslipidemia, were increased when the effects of two types of excess weight were combined. According to the data from the China Health and Nutrition Survey (CHNS), more than 65% of individuals with obesity would be missed if BMI alone was measured [8]. Therefore, we believe that efforts to promote the measurement of WC in clinical practice are crucial.

The present study has several limitations. The major one is its cross-sectional nature, which suggests that it cannot provide sufficient evidence of causality. Second, China is a vast country with diverse lifestyles. Our data are not representative of the rural population throughout the country and the findings cannot be extrapolated to other provinces. In addition, although the researchers had been trained according to a standardized protocol of measurements, measurements at a single visit might lead to incorrect values for the anthropometric indexes. Also, the sample size of ethnic minorities was too small and the conclusion should be carefully considered. Other limitations include recall bias and the small sample sizes available in some other subgroup analyses that reduce the statistical power to detect significant associations.

## Conclusions

In summary, our population-based study indicated a dramatically increased prevalence of overweight and obesity in rural areas of Northeast China. General and abdominal obesity exhibited different risk profiles and were related to different adverse comorbidities. The large obese population and the wide-ranging effects that excess body weight has on health should be considered in the development of rural areas. The combined effects of both obesity types on cardiometabolic comorbidities were much greater than either one alone, indicating the importance of the combined use of both WC and BMI in clinical work.

## Abbreviations

CVD: Cardiovascular disease
BMI: Body mass index
WC: Waist circumference
BP: Blood pressure
FPG: Fasting plasma glucose
TC: Total cholesterol
LDL-C: Low-density lipoprotein cholesterol
HDL-C: High-density lipoprotein cholesterol
TG: Triglyceride
SBP: Systolic BP
DBP: Diastolic BP
WHO: World Health Organization
OR: Odds ratio
CI: Confidence interval.
